# Adsorption and Catalytic Reduction of Nitrogen Oxides (NO, N_2_O) on Disulfide Cluster Complexes of Cobalt and Iron—A Density Functional Study

**DOI:** 10.3390/ma17194764

**Published:** 2024-09-28

**Authors:** Ellie L. Uzunova, Ivelina M. Georgieva

**Affiliations:** Institute of General and Inorganic Chemistry, Bulgarian Academy of Sciences, 1113 Sofia, Bulgaria; ivelina@svr.igic.bas.bg

**Keywords:** ab initio methods, DFT, transition metal sulfides, carbonyl complexes, nitrosyls

## Abstract

The reactivity of nitrogen oxide, NO, as a ligand in complexes with [Fe2-S2] and [Co2-S2] non-planar rhombic cores is examined by density functional theory (DFT). The cobalt-containing nitrosyl complexes are less stable than the iron complexes because the Co-S bonds in the [Co2-S2] core are weakened upon NO coordination. Various positions of NO were examined, including its binding to sulfur centers. The release of NO molecules can be monitored photochemically. The ability of NO to form a (NO)_2_ dimer provides a favorable route of electrochemical reduction, as protonation significantly stabilizes the dimeric species over the monomers. The quasilinear dimer ONNO, with trans-orientation of oxygen atoms, gains higher stability under protonation and reduction via proton–electron transfer. The first two reduction steps lead to an N_2_O intermediate, whose reduction is more energy demanding: in the two latter reaction steps the highest energy barrier for Co_2_S_2_(CO)_6_ is 109 kJ mol^−1^, and for Fe_2_S_2_(CO)_6_, it is 133 kJ mol^−1^. Again, the presence of favorable light absorption bands allows for a photochemical route to overcome these energy barriers. All elementary steps are exothermic, and the final products are molecular nitrogen and water.

## 1. Introduction

Nitrogen oxides are produced mainly by vehicles, but also by coal power stations. They are harmful to the environment, contributing to acid rain, and pose a danger to human health [1,2]. There is very limited direct usage of NO and N_2_O, as is the case with CO_2_. NO and N_2_O find application in medicine, with N_2_O being used as an anesthetic, while the nitrosyl ligand in diiron–disulfur [Fe2-S2] and dicobalt–disulfur [Co2-S2] complexes proved efficient to deliver NO to targeted cells [3,4,5,6]. The active core [Fe2-S2] is made of natural ferredoxin and hydrogenase enzymes, with the coordination of carbonyl and cyanide ligands [7,8]. Whether coordinated by nitrosyl, or by carbonyl ligands, the [Fe2-S2] and [Co2-S2] complexes possess photo-reactivity [3,4,5,6,7,8,9]. The chemistry and photochemistry of nitrosyl and carbonyl complexes differ considerably. Up to six carbonyl ligands can be coordinated to a [Fe2-S2] core, but the maximum coordination number for nitrosyl groups is four [3,5,9]. The tetra-nitrosyls are less stable than the corresponding carbonyl complexes, and the cobalt-containing nitrosyls are less stable than their iron analogs [3,5]. In carbonyl complexes, the release of carbonyl ligands is energy-demanding, while in nitrosyl complexes, the NO ligands have smaller binding energies and thus are more easily released. The nitrogen oxide molecule, NO, is more reactive than CO and it forms a dimer, O-N-N-O, in two conformations: with cis-orientation of oxygen atoms and with trans-orientation [10,11,12]. The cis-configuration is the global minimum, though it has a strongly lengthened N-N bond [10]. Experimental studies proved the co-existence of the monomer, NO, and the dimer, (NO)_2_, in gaseous nitrogen oxide, [11]. Despite the higher reactivity of NO and N_2_O as compared to CO_2_, in the selective catalytic reduction of NO_x_, most often carbon monoxide (CO), or a hydrocarbon, such as propane, is used as a reducing agent over a broad range of catalyst materials: transition metal sulfides [13] and transition metal surfaces [14]. Novel approaches with hydrogen as a reducing agent as an eco-friendly alternative were also proposed [15]. Irrespective of the reducing compound used, N_2_O always appears as an intermediate product. The direct decomposition of N_2_O, without a co-reactant, was studied on transition metal oxides [16] and transition metal cation-exchanged zeolites, [17]. Transition metal sulfide complexes, with their powerful redox catalyst capacity, boosted by photo-reactivity, are good candidates for NO electro-photochemical redox catalysts.

The present theoretical study explores the electrocatalytic reduction of NO with the hexacarbonyl complexes Fe_2_S_2_(CO)_6_ and Co_2_S_2_(CO)_6_, already known as water-splitting electro-photocatalysts. The dimer (NO)_2_ is used as a reactant for the reaction of dissociation to N_2_ and O_2_, as it has much higher proton and proton–electron (H^+^,e^−^) affinities compared to the monomer NO. The reaction mechanism is followed by transition state theory. As the monomer NO is also present in real conditions, the interaction and coordination of NO to the complexes were also studied. The substitution of CO by NO as a ligand in Fe_2_S_2_(CO)_6_ and Co_2_S_2_(CO)_6_ is examined at different levels of substitution, by one, two, and up to four NO groups, provided that the resulting complexes remain stable. All possible coordination sites for NO are considered, including its binding to a sulfur center. The structures and electron distributions are determined by density functional theory (DFT) and compared to experimental data, where available. The mixed carbonyl–nitrosyl complexes are also studied by time-dependent DFT (TD-DFT) to reveal the excitation bands, favoring NO release by these complexes.

## 2. Materials and Methods

The calculations in the present study are performed with density functional theory methods as implemented in Gaussian 16 [18]. Details on basis sets and methods are presented in Supplementary Materials. In their global minima, Fe_2_(S_2_)(CO)_6_ is diamagnetic, Co_2_(S_2_)(CO)_6_ is antiferromagnetic, and both complexes have a non-planar core with bridging disulfur (see Figure S1 in Supplementary Materials). Though the two complexes have different magnetic structures, the attachment of proton–electron coupling, [H^+^,e^−^], during the path of electrochemical reduction, changes the electronic state of both hexacarbonyl complexes to a doublet state. The nitrogen oxide dimer (NO)_2_ exists in two isomeric forms: the cis form, with a lengthened N-N bond and the two oxygen atoms on one side; and a quasilinear trans-form, with the two oxygen atoms on different sides of the N-N bond (see Table 1 and Figure S2 in Supplementary Materials). The nitrogen oxide molecule attaches a proton at the N-center, while in N_2_O, the protons may be attached at both ends. N_2_O has a higher proton affinity than NO, and the preferred position for protonation is the oxygen atom. The attachment of a proton–electron couple to N_2_O, however, leads to hydroxyl group dissociation even without the presence of a catalyst. The NO dimer, (NO)_2_, has a much higher proton affinity and proton–electron affinity, as compared to all other NOx species. The protonation renders the trans-quasilinear isomer ONNO as more stable than the cis-isomer by 42 kJ mol^−1^, as its proton affinity is much higher. The proton–electron affinity [H^+^,e^−^] of ONNO is also higher. These results indicate that the nitrosyl dimer, ONNO, is a good candidate for starting the process of catalytic reduction.

## 3. Results and Discussion

### 3.1. Binding of NO to the Carbonyl Complexes Fe_2_S_2_(CO)_6_ and Co_2_S_2_(CO)_6_

**Figure 1:**
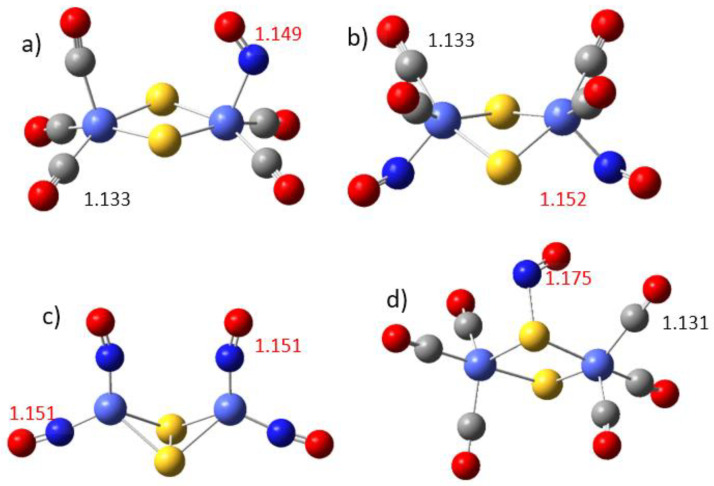
Mixed nitrosyl–carbonyl and pure nitrosyl complexes: (**a**) Co_2_S_2_(CO)_5_(NO); (**b**) Co_2_S_2_(CO)_4_(NO)_2_; (**c**) Co_2_S_2_(NO)_4_; and (**d**) Co_2_S_2_(CO)_6_(NO) with S-NO bond. Legend: cobalt cations are light-blue large balls, sulfur atoms are yellow, nitrogen is dark blue, oxygen is red, and carbon is gray. N-O bond lengths are marked red, and C-O bond lengths are black. The M-S bonds, M-N bonds, and S-N bonds are described in Table 2. The corresponding Fe complexes are presented in Figure S3 in Supplementary Materials.

The nitrosyl ligand can bind either directly to the metal cation at the place of one or two carbonyl groups; it can also form an entire nitrosyl complex with a maximum of four nitrosyl groups, Fe_2_S_2_(NO)_4_ and Co_2_S_2_(NO)_4_ as shown in Figure 1, which were synthesized experimentally [3,4,5]. Our calculations indicate that nitrosyl binding to a sulfur site is also possible in the hexacarbonyls (see Figure 1c) though with significantly lower binding energy (Table 2). The binding of nitrosyl groups to dicobalt–disulfide is considerably weaker than the binding to the diiron–disulfide complex, in agreement with experimental results [5]. In the dicobalt–disulfide tetra-nitrosyl complex Co_2_S_2_(NO)_4_, the Co-N bonds are slightly lengthened, by 0.005 Å, as compared to Fe_2_S_2_(NO)_4_, but the Co-S bonds are lengthened more significantly, by 0.149 Å. The coordination of nitrosyl groups in all cases breaks the S-S bond, present in the global minima of the hexacarbonyl complexes. Natural bond orbital analysis (NBO) reveals that the N-O bonds are less polarized than the C-O bonds. The local natural charge on N is +0.342, and −0.152 on O of the nitrosyl, whereas it is +0.815 on C and −0.426 on O of the CO ligand. The nitrosyl groups acquire some spin density of the order 0.3 to 0.4, while the carbonyl groups acquire negligible spin density.

All of the nitrosyl complexes have their most intense light absorption bands in the visible region of the spectrum. In the mixed carbonyl–nitrosyl complexes with the nitrosyl groups coordinating the metal cations, the bands of the diiron–disulfide complex are red-shifted compared to the dicobalt–disulfide complex (Table 3). In the complexes with S-NO bonds, the most intense absorption bands of the dicobalt–disulfide complex become red-shifted to 820 nm. The nitrosyl ligands are weakly bonded, as compared to carbonyl ligands—the release of a carbonyl group from Fe_2_S_2_(CO)_6_ requires 154 kJ mol^−1^ [20]. The highest energy needed for nitrosyl group release is found in the tetra-nitrosyl of iron Fe_2_S_2_(NO)_4_, at 88.5 kJ mol^−1^. The excitation band at 535 nm and even the near-IR band at 1057 nm provide enough energy for the release of nitrosyl groups in this stable complex. The bands in the visible region of the spectrum have a dominant Metal-to-Ligand Charge Transfer (MLCT) character, and electron transfer to the nitrosyl ligand occurs.

### 3.2. Reduction of NO in the Form of Its Dimer, (NO)_2_

The strong proton affinity of the quasilinear trans-ONNO, 682 kJ mol^−1^, comparable to the proton affinity of Fe_2_(S_2_)(CO)_6_, 717 kJ mol^−1^, renders the nitrosyl dimer as a promising reactant for selective electrocatalytic reduction in the following reaction scheme:

M_2_S_2_(CO)_6_ + H^+^,e^−^ + ONNO ⇒ M_2_S_2_(CO)_6_[ H^+^,e^−^] + ONNO ⇒ M_2_S_2_(CO)_6_ + ONNOH^∙^ (1)

M_2_S_2_(CO)_6_ + ONNOH^∙^ + H^+^,e^−^ ⇒ M_2_S_2_(CO)_6_[ H^+^,e^−^] + ONNOH^∙^ ⇒ M_2_S_2_(CO)_6_ + N_2_O + H_2_O (2)

M_2_S_2_(CO)_6_ + N_2_O + H^+^,e^−^ ⇒ M_2_S_2_(CO)_6_[H^+^,e^−^] + N_2_O ⇒ M_2_S_2_(CO)_6_ + N_2_ + OH^∙^ (3)

M_2_S_2_(CO)_6_ + H^+^,e^−^ + OH^∙^ ⇒ M_2_S_2_(CO)_6_[H^+^,e^−^][OH^∙^] ⇒ M_2_S_2_(CO)_6_ +H_2_O (4)

**Figure 2:**
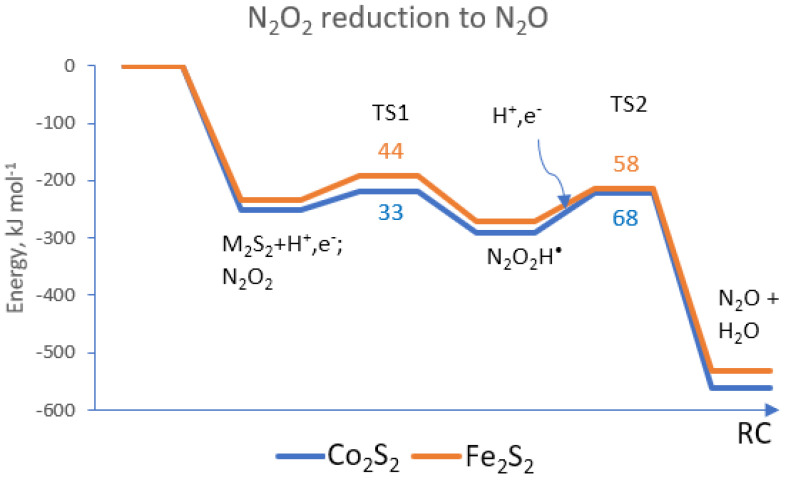
The reaction path of N_2_O_2_ dissociation to N_2_O on Co_2_S_2_(CO)_6_ and Fe_2_S_2_(CO)_6_. TS1 denotes the energy barrier of reaction step (1) and TS2is the energy barrier of elementary step (2). RC—reaction coordinate.

Steps (1) and (2) lead to N_2_O, and at this stage, Co_2_S_2_(CO)_6_ and Fe_2_S_2_(CO)_6_ perform equally well, with a small difference in the energy barriers of 10–11 kJ mol^−1^ (see Figure 2). For the first step of N_2_O_2_ hydrogenation, the cobalt complex Co_2_S_2_(CO)_6_ provides a low energy barrier of only 33 kJ mol^−1^; for the iron complex, the energy barrier is only 11 kJ mol^−1^ higher, at 44 kJ mol^−1^. This reaction step is weakly exothermic, at 38 kJ mol^−1^ for the cobalt complex, and at 36 kJ mol^−1^ for the iron complex—the values are again very close. The reaction barrier of the second elementary step (2) to the formation of N_2_O and release of a water molecule is about twice as high for the cobalt complex (68 kJ mol^−1^) as compared to the first step. For this step, the iron complex provides a similar, slightly lower energy barrier.

**Figure 3:**
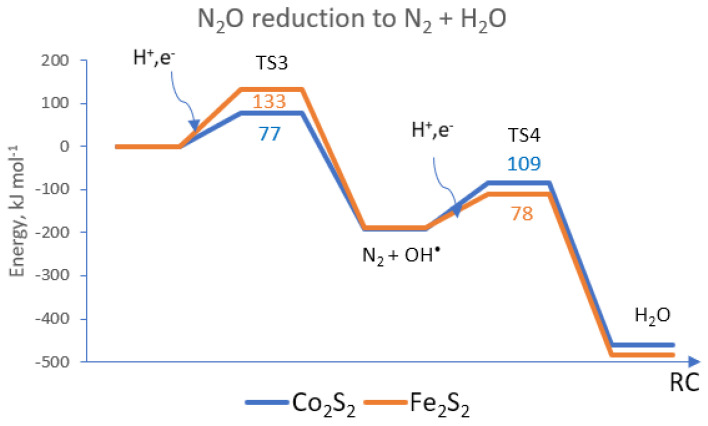
The reaction path of N_2_O dissociation to N_2_ and H_2_O on hexacarbonyl complexes with Co_2_S_2_ and Fe_2_S_2_ core. TS3 denotes the energy barrier to reaction step (3) and TS4 is the energy barrier to elementary step (4). RC—reaction coordinate.

**Figure 4:**
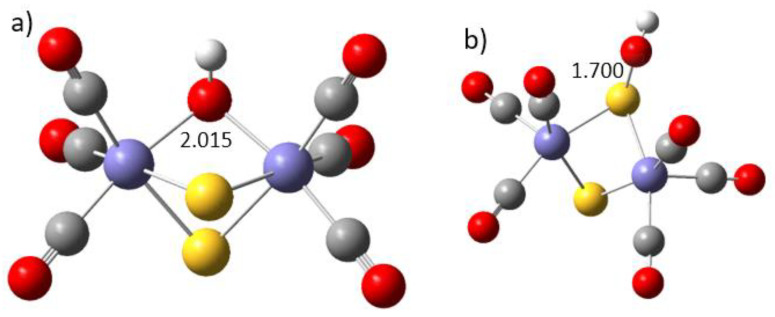
The binding of the OH^∙^ group to Fe_2_S_2_(CO)_6_. (**a**) Midway position of the OH^∙^ group bonded directly to the Fe centers; (**b**) OH^∙^ group bonded to the sulfur atom. Configuration (**a**) is the global minimum, found as 30 kJ mol^−1^ below configuration (**b**). Iron cations are large aqua-blue balls, sulfur atoms are yellow, nitrogen is dark blue, oxygen is red, and carbon is gray. The cobalt complex is presented in Figure S7 in Supplementary Materials.

The subsequent reduction of N_2_O proceeds via elementary steps (3) and (4) and yields molecular nitrogen and water. For reaction step 3, the Co_2_S_2_(CO)_6_ complex provides a much lower energy barrier of 77 kJ mol^−1^ vs. 133 kJ mol^−1^ for the Fe_2_S_2_(CO)_6_ complex (Figure 3). In this step, molecular nitrogen is formed and an OH^∙^ group is released, which becomes attached to the disulfide core of the complexes. Step (3) is thus strongly exothermic, as it leads to the release of molecular nitrogen, together with the formation of a hydroxyl-bonded disulfide complex (Figure 4). Only Fe_2_S_2_(CO)_6_ provides two alternative locations of the OH^∙^ group: a midway position, attached to both iron centers symmetrically via the oxygen atom of OH^∙^; and coordination to a sulfur atom, with the formation of a relatively short S-O bond (Figure 4b). In the Co_2_S_2_(CO)_6_ complex, the OH^∙^ group is allowed to bind to the sulfur center only; see Figure S7 in Supplementary Materials. The final step (4) of the ONNO reduction reaction is the release of a water molecule to restore the catalyst center. In this step, the diiron–disulfide complex has a lower energy barrier of 78 kJ mol^−1^, which is possibly due to the flexibility of the OH^∙^ group binding. The energy barrier of the dicobalt–disulfide complex is a bit higher, at 109 kJ mol^−1^.

**Figure 5:**
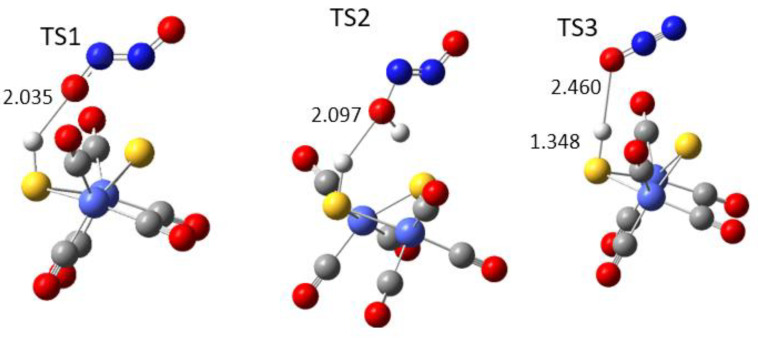
Transition state structures along the reaction path of ONNO reduction for the [Co2-S2] complex. TS1 reveals the first reduction step of ONNO. TS2 corresponds to the reduction of ONNOH^∙^. TS3 refers to the reduction of N_2_O. Legend is the same as Figure 1. The transition states TS1, TS2, TS3, and TS4 (water molecule release) for the [Fe2-S2] complex are presented as Supplementary Materials in Figure S7, and their coordinates are included.

The reaction route is exothermic in all four elementary steps. Transition states 1 and 2 are closer to the configuration of the initial reactants than to the reaction products. TS3 is about midway in the route to the final products in this step: the release of the N_2_ and OH^∙^ groups (see Figure 5). For the cobalt complex, N_2_O is linear in TS3 and is closer to the initial state, while the iron complex reaches TS3 upon bending the N_2_O molecule, resulting in a much higher energy barrier.

Though the highly exothermic elementary steps may compensate for the subsequent high energy barriers, the possibility of performing a photocatalyzed reaction was also examined in Table 3. The OH^•^ group containing complexes possess strongly intense bands in the visible spectral region, particularly for isomers with S-OH bonds, and they are thus capable of providing the necessary energy for the barrier in step (4) and restoring the catalyst to its initial state.

## 4. Conclusions

The reactivity of nitrogen oxide, NO, was examined as a ligand in carbonyl complexes with [Fe2-S2] and [Co2-S2] non-planar rhombic cores. By increasing the number of nitrosyl ligands, the M-N bond is strengthened, particularly in the iron complexes, but the release of NO can be achieved photochemically. The dimerization of NO opens up a possibility for a low-energy pathway of electrocatalytic reduction. The greatest advantage over other reaction schemes is that there is no oxygen abstraction, but water formation occurs in the elementary steps. The highest energy barriers are reached in the reduction of N_2_O, which appears as an intermediate product, but for Co_2_S_2_(CO)_6_, the highest energy barrier is 109 kJ mol^−1^, and for Fe_2_S_2_(CO)_6_, it is 133 kJ mol^−1^. The presence of favorable light absorption bands in the visible spectrum provides an opportunity for photocatalyzed electrochemical reduction.

## Figures and Tables

**Table 1:** Bond lengths, proton affinities (PAs), and H^+^,e^−^ affinities of nitrogen oxides: NO, N_2_O, and the NO dimer, N_2_O_2_.

Molecule	Bond Length N-O, Å	Bond Length N-N, Å	PA, kJ mol^−1^	H^+^,e^−^ Affinity, kJ mol^−1^
NO	1.1456		529	233
Exp, Ref. [18]	1.1508		531	
N_2_O	1.1840	1.1210	554 at N 582 at O	
Exp, Ref. [19]	1.1860	1.1260	549.8 at N 575.2 at O	
(NO)_2_ cis, N_2_O_2_	1.1471	1.9730	638	286
(NO)_2_ trans-ONNO	1.2118	1.1564	682	311

**Table 2:** Selected bond lengths in mixed carbonyl–nitrosyl complexes with M2S2 core (M = Fe, Co) and nitrosyl group binding energies (BEs) at different sites.

Molecule	Bond Length M-S, Å	Bond Length M-N, Å	Bond Length S-N, Å	NO BE, kJ mol^−1^
Co_2_S_2_(CO)_5_(NO) Co-NO bond	2.236	1.964		17.4
Co_2_S_2_(CO)_4_(NO)_2_ 2 Co-NO bonds	2.245	1.821		30.1
Co_2_S_2_(NO)_4_	2.317	1.641		76.1
Co_2_S_2_(CO)_6_(NO) S-NO bond	2.297; 2.373		1.864	22.4
Fe_2_S_2_(CO)_5_(NO) Fe-NO bond	2.236	1.726		50.4
Fe_2_S_2_(CO)_4_(NO)_2_ 2 Fe-NO bonds	2.275	1.722		58.6
Fe_2_S_2_(NO)_4_	2.168	1.636		88.5
Fe_2_S_2_(CO)_6_(NO) S-NO bond	2.231; 2.273		1.939	32.1

**Table 3:** TD-DFT results for nitrosyl and hydroxyl complexes. The most intense lines are listed. UV-VIS spectra are illustrated in Supplementary Materials in Figures S4–S6.

Complex, Bonds	Light Absorption, nm	Oscillator Strength
Co_2_S_2_(CO)_5_(NO) Co-NO bond	663	0.0025
Fe_2_S_2_(CO)_5_(NO) Fe-NO bond	739	0.0065
Co_2_S_2_(NO)_4_	524	0.0174
Fe_2_S_2_(NO)_4_	535 1057	0.0011 0.0087
Co_2_S_2_(CO)_6_(NO) S-NO bond	820	0.0133
Fe_2_S_2_(CO)_6_(NO) S-NO bond	739	0.0065
Co_2_S_2_(CO)_6_(OH) S-OH bond	874	0.0282
Fe_2_S_2_(CO)_6_(OH) S-OH bond	965	0.0205
Fe_2_S_2_(CO)_6_(OH) Fe-OH-Fe bond	679	0.0091

## Data Availability

The original contributions presented in the study are included in the article/Supplementary Materials, further inquiries can be directed to the corresponding author.

## Supplementary Materials

The following supporting information can be downloaded at https://www.mdpi.com/article/10.3390/ma17194764/s1: Computational details: structures of complexes and reactants; Figures S1–S3; UV-VIS spectra; Figures S4–S6: Transition state structures and intermediates; Figures S7 and S8: Transition state structures and their coordinates. References [21,22,23,24,25,26,27,28,29,30,31,32,33,34,35,36,37,38,39,40] are cited in the Supplementary Materials.

## References

[1] Leclercq B., Kluza J., Antherieu S., Sotty J., Alleman L.Y., Perdrix E., Loyens A., Coddeville P., Lo Guidice J.-M., Marchetti P. (2018). Air pollution-derived PM2.5 impairs mitochondrial function in healthy and chronic obstructive pulmonary diseased human bronchial epithelial cells. Environ. Pollut..

[2] Anenberg S.C., Miller J., Minjares R., Du L.I., Henze D.K., Lacey F., Malley C.S., Emberson L., Franco V., Klimont Z. (2017). Impacts and mitigation of excess diesel-related NOx emissions in 11 major vehicle markets. Nature.

[3] Bourassa J., DeGraff W., Kudo S., Wink D.A., Mitchell J.B., Ford P.C. (1997). Photochemistry of Roussin’s Red Salt, Na_2_[Fe_2_S_2_(NO)_4_], and of Roussin’s Black Salt, NH_4_[Fe_4_S_3_(NO)_7_]. In Situ Nitric Oxide Generation to Sensitize *γ*-Radiation Induced Cell Death. J. Am. Chem. Soc..

[4] Harrop T.C., Tonzetich Z.J., Reisner E., Lippard S.J. (2008). Reactions of Synthetic [2Fe-2S] and [4Fe-4S] Clusters with Nitric Oxide and Nitrosothiols. J. Am. Chem. Soc..

[5] Bitterwolf T.E., Pal P. (2006). Synthesis of the cobalt analogues of Roussin’s red salt esters Inorg. Chim. Acta.

[6] Hayton T.W., Legzdins P., Sharp W.B. (2002). Coordination and Organometallic Chemistry of Metal-NO Complexes. Chem. Rev..

[7] Tard C., Pickett C.J. (2009). Structural and Functional Analogues of the Active Sites of the [Fe]-, [NiFe]-, and [FeFe]-Hydrogenases. Chem. Rev..

[8] Appel A.M., Bercaw J.E., Bocarsly A.B., Dobbek H., DuBois D.L., Dupuis M., Ferry J.G., Fujita E., Hille R., Kenis P.J.A. (2013). Frontiers, Opportunities, and Challenges in Biochemical and Chemical Catalysis of CO_2_ Fixation. Chem. Rev..

[9] Nann T., Ibrahim S.K., Woi P.-M., Xu S., Ziegler J., Pickett C.J. (2010). Water Splitting by Visible Light: A Nanophotocathode for Hydrogen Production. Angew. Chem. Int. Ed..

[10] Harcourt R.D. (1990). The origin of the long N-N bond in N_2_O_2_: An ab initio valence bond study. J. Mol. Str.: THEOCHEM.

[11] Dkhissi A., Soulard P., Perrin A., Lacome N. (1997). “The NO Dimer”. J. Mol. Spectrosc..

[12] East A.L.L. (1998). The 16 valence electronic states of nitric oxide dimer. J. Chem. Phys..

[13] Esrafili M.D., Janebi H., Mousavian P. (2021). Single Al atom anchored on defective MoS2: An efficient catalytic site for reduction of greenhouse N_2_O gas by CO or C_2_H_4_ molecules. Appl. Surf. Sci..

[14] Matsushima T., Kokalj A. (2017). N2 emission in steady-state N2O + CO and NO + CO reactions on Ir(110) by means of angle-resolved desorption. Appl. Surf. Sci..

[15] Farhan S.M., Pan W., Zhijian C., JianJun Y. (2024). Innovative catalysts for the selective catalytic reduction of NO_x_ with H_2_: A systematic review. Fuel.

[16] Kocía K., Relia M., Troppováa I., Sihor M., Kupkovác J., Kustrowski P., Prausa P. (2017). Photocatalytic decomposition of N2O over TiO_2_/g-C_3_N_4_ photocatalysts heterojunction. Appl. Surf. Sci..

[17] Ryder J.A., Chakraborty A.K., Bell A.T. (2002). Density Functional Theory Study of Nitrous Oxide Decomposition over Fe- and Co-ZSM-5. J. Phys. Chem. B.

[18] Kuo S.C., Zhang Z.Y., Ross S.K., Klemm R.B., Johnson R.D., Monks P.S., Thorn R.P., Stief L.J. (1997). Discharge flow-photoionization mass spectrometric study of HNO: Phtoionization efficiency spectrum and ionization energy and proton affinity of NO. J. Phys. Chem. A.

[19] Hunter E.P., Lias S.G. (1998). Evaluated Gas Phase Basicities and Proton Affinities of Molecules: An Update. J. Phys. Chem. Ref. Data.

[20] Uzunova E.L., Mikosch H. (2014). Electronic, magnetic structure and water splitting reactivity of the iron-sulfur dimers and their hexacarbonyl complexes: A density functional study. J. Chem. Phys..

[21] Becke A.D. (1996). Density-functional thermochemistry. IV. A new dynamical correlation functional and implications for exact-exchange mixing. J. Chem. Phys..

[22] Becke A.D. (1993). Density-functional thermochemistry. *III. The role of exact exchange*. J. Chem. Phys..

[23] Lee C., Yang W., Parr R.G. (1988). Development of the Colle-Salvetti correlation-energy formula into a functional of the electron density. Phys. Rev. B.

[24] Miehlich B., Savin A., Stoll H., Preuss H. (1989). Results obtained with the correlation energy density functionals of Becke and Lee, Yang and Parr. Chem. Phys. Lett..

[25] Wachters A.J.H. (1970). Gaussian basis set for molecular wavefunctions containing third-row atoms. J. Chem. Phys..

[26] Hay P.J. (1977). Gaussian basis sets for molecular calculations. The representation of 3d orbitals in transition-metal atoms. J. Chem. Phys..

[27] Raghavachari K., Trucks G.W. (1989). Highly correlated systems. Excitation energies of first row transition metals Sc–Cu. J. Chem. Phys..

[28] Hay P.J., Wadt W.R. (1985). Ab initio effective core potentials for molecular calculations. Potentials for the transition metal atoms Sc to Hg. J. Chem. Phys..

[29] Halgren T.A., Lipscomb W.N. (1977). The synchronous-transit method for determining reaction pathways and locating molecular transition states. Chem. Phys. Lett..

[30] Peng C., Ayala P.Y., Schlegel H.B., Frisch M.J. (1996). Using redundant internal coordinates to optimize equilibrium geometries and transition states. J. Comp. Chem..

[31] Fukui K. (1981). The path of chemical reactions-the IRC approach. Acc. Chem. Res..

[32] Hratchian H.P., Schlegel H.B. (2004). Accurate reaction paths using a Hessian based predictor–corrector integrator. J. Chem. Phys..

[33] Bauernschmitt R., Ahlrichs R. (1996). Stability analysis for solutions of the closed shell Kohn–Sham equation. J. Chem. Phys..

[34] Bauernschmitt R., Ahlrichs R. (1996). Treatment of electronic excitations within the adiabatic approximation of time dependent density functional theory. Chem. Phys. Lett..

[35] Furche F., Ahlrichs R. (2002). Adiabatic time-dependent density functional methods for excited state properties. J. Chem. Phys..

[36] Yanai T., Tew D., Handy N. (2004). A new hybrid exchange-correlation functional using the Coulomb-attenuating method (CAM-B3LYP). Chem. Phys. Lett..

[37] Reed A.E., Curtiss L.A., Weinhold F. (1988). Intermolecular interactions from a natural bond orbital, donor-acceptor viewpoint. Chem. Rev..

[38] Weinhold F., Carpenter J.E. (1988). The Structure of Small Molecules and Ions.

[39] Tomasi J., Mennucci B., Cammi R. (2005). Quantum Mechanical Continuum Solvation Models. Chem. Rev..

[40] Grimme S., Ehrlich S., Goerigk L. (2011). Effect of the damping function in dispersion corrected density functional theory. J. Comp. Chem..

