# Supplementary material for: Adsorption and Catalytic Reduction of Nitrogen Oxides (NO, N2O) on Disulfide Cluster Complexes of Cobalt and Iron—A Density Functional Study
Source: Materials (Basel). 2024 Sep 28;17(19):4764. doi: 10.3390/ma17194764 (PMC11477673; doi:10.3390/ma17194764)
Supplement: Supplementary file 1 [file materials-17-04764-s001.zip › materials-3198907-supplementary.pdf]

Supplementary

Adsorption and Catalytic Reduction of Nitrogen Oxides (NO, N_2O) on Disulfide Cluster Complexes of Cobalt and Iron—A Density Functional Study

Ellie Uzunova * and Ivelina Georgieva

Institute of General and Inorganic Chemistry, Bulgarian Academy of Sciences, 1113 Sofia, Bulgaria; ivelina@svr.igic.bas.bg
* Correspondence: ellie@svr.igic.bas.bg

Computational Details

The B3LYP density functional was used throughout this study. [1–4] All calculations were performed in the spin-unrestricted formalism. The standard basis set 6-311+G(2df) with diffuse and polarization functions was employed and polarization functions for hydrogen were also included. This is a Wachters-Hay all electron basis set for the first transition row, using the scaling factors of Raghavachari and Trucks. [5–8] In terms of atomic orbitals, the basis set is represented as [10s7p4d2f1g] for iron and cobalt, [7s6p2d1f] for sulfur, [5s4p2d1f] for nitrogen, oxygen and carbon, and [3s3p] for hydrogen. The synchronous transit-guided quasi-Newton (STQN) method was used for the transition state optimizations. [9,10] Intrinsic reaction coordinate calculations (IRC) were performed to confirm the structure of the transition states and for evaluating activation energies. [11,12] The vertical excitation energies of metal-disulfide coordination compounds and their reactive (H^+,e^-) forms to the low-lying singlet and triplet states were determined by time-dependent (TD) DFT. [13–15] The CAM-B3LYP density functional was employed for calculation of light absorption bands in the UV-VIS spectra and for these computations, the geometries of the compounds were reoptimized at the CAM-B3LYP level (CAM- the Coulomb attenuated modification of B3LYP) [16] with the basis sets mentioned above. The bond populations and charge distributions were examined by using natural orbitals and natural bond orbital (NBO) analysis. [17,18] The energy of proton-electron couples is $E(H^+,e^-)$ = 0.502156 Hartree, calculated as a free hydrogen atom. The attachment of each proton-electron couple (H^+,e^-) is performed in the sequence (i) proton attachment, followed by (ii) electron attachment. Reaction studies using water as a solvent were performed using the Polarizable Continuum Model (PCM) [19]. Dispersion effects were taken into account for the ground states and the reaction intermediates by using the formula of Grimme with Becke-Johnson damping [20].

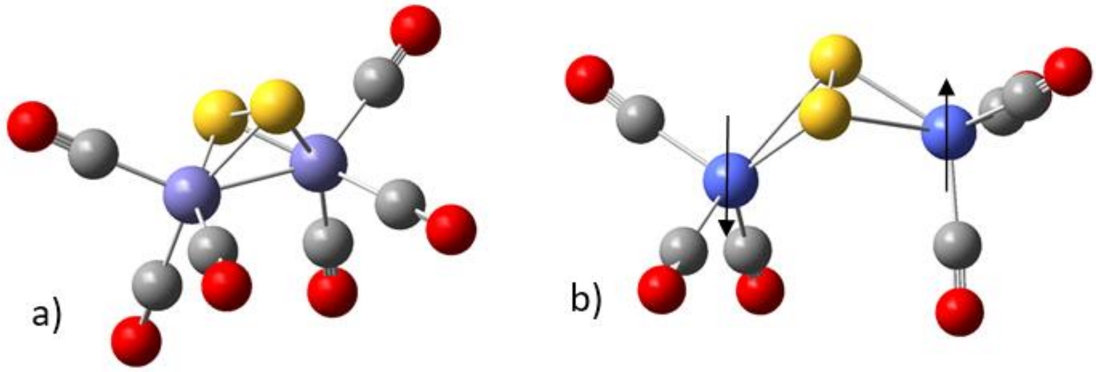

Figure S1. Global minima of hexacarbonyl complexes with [Fe_2S_2] and [Co_2S_2] core: a) Diamagnetic ground state of $(CO)_6Fe_2$-μ-η^2:η^2-S_2, denoted for simplicity further as $Fe_2(S_2)(CO)_6$ b)

Antiferromagnetic ground state of $(CO)_6Co_2$-μ-η^2:η^2-S_2, denoted further as $Co_2(S_2)(CO)_6$. Iron cations are aqua-blue large balls, cobalt cations are light-blue, sulfur atoms are yellow, oxygen – red, and carbon – grey. The black arrows denote the location of unpaired electrons and the spin orientation.

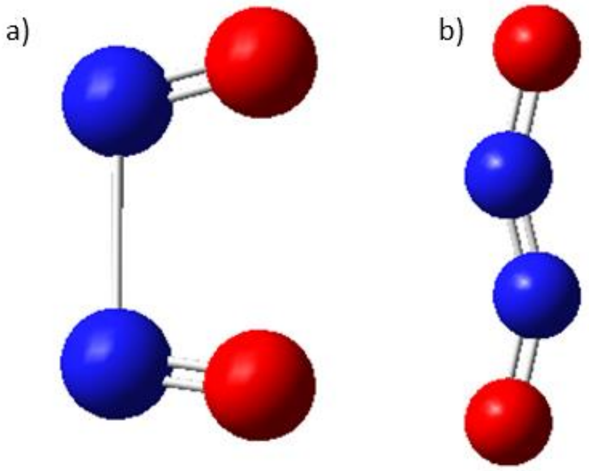

Figure S2. the structure of the NO dimer: a) the global minimum, cis isomer, b) the quasilinear trans-isomer. Bond lengths denoted in Table 1 of main text. Nitrogen atoms are dark-blue, oxygen is red.

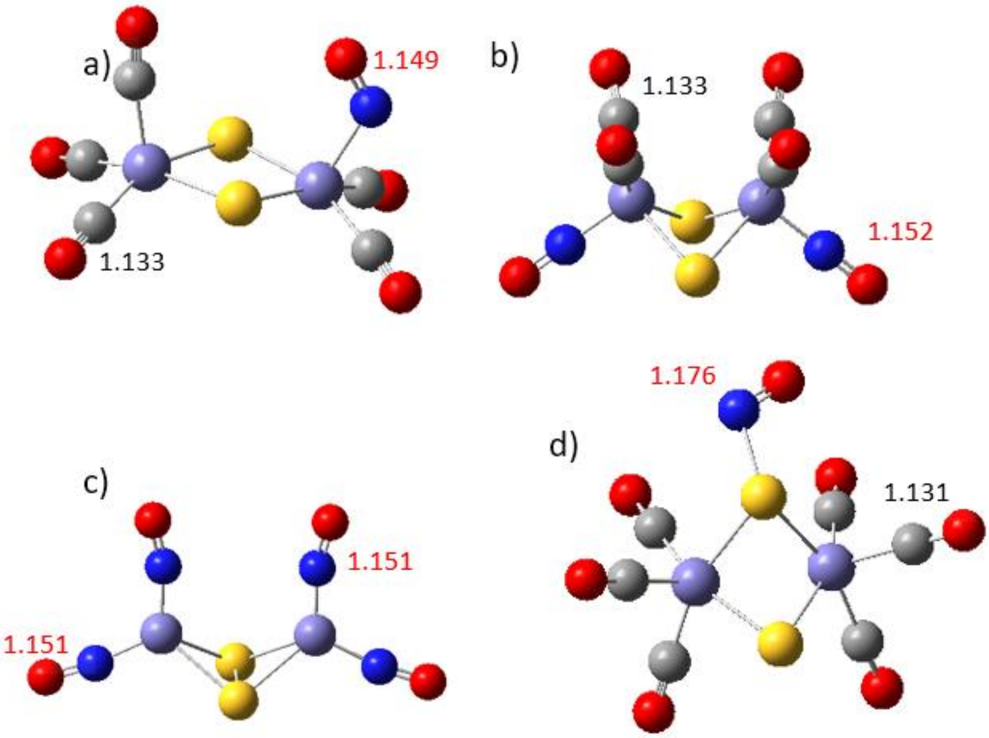

Figure S3. Mixed nitrosyl-carbonyl and pure nitrosyl complexes: a) $Fe_2S_2(CO)_5(NO)$; b) $Fe_2S_2(CO)_4(NO)_2$ c) $Fe_2S_2(NO)_4$; d) $Fe_2S_2(CO)_6(NO)$ with S-NO bond. Legend: Transition metal cations are light-blue large balls, sulfur atoms are yellow, nitrogen – dark blue, oxygen – red, and carbon – grey. N-O bond lengths are marked red, C-O bond lengths are black. M-S bonds, M-N bonds and S-N bonds are described in Table 2 of the main text.

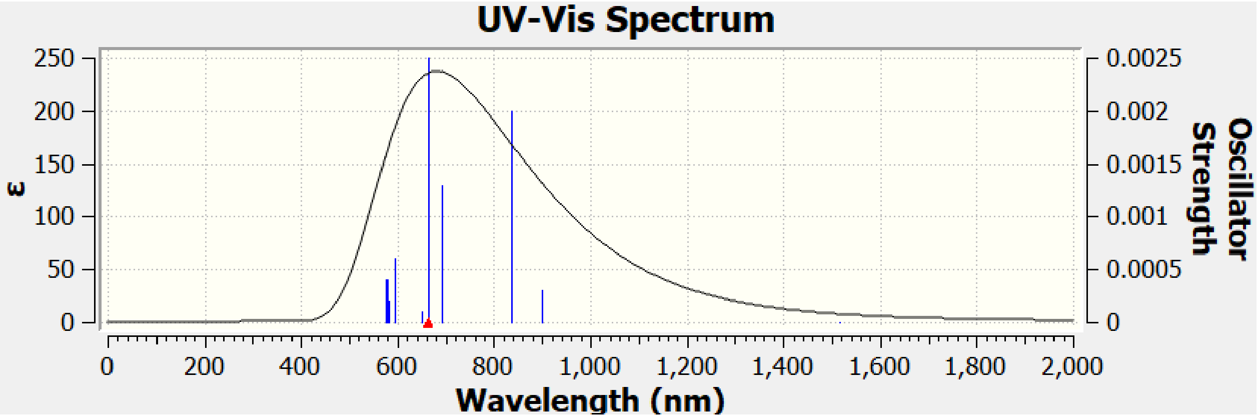

Figure S4. UV-VIS spectrum of $Co_2S_2(CO)_5(NO)$ with Co-(NO) bond.

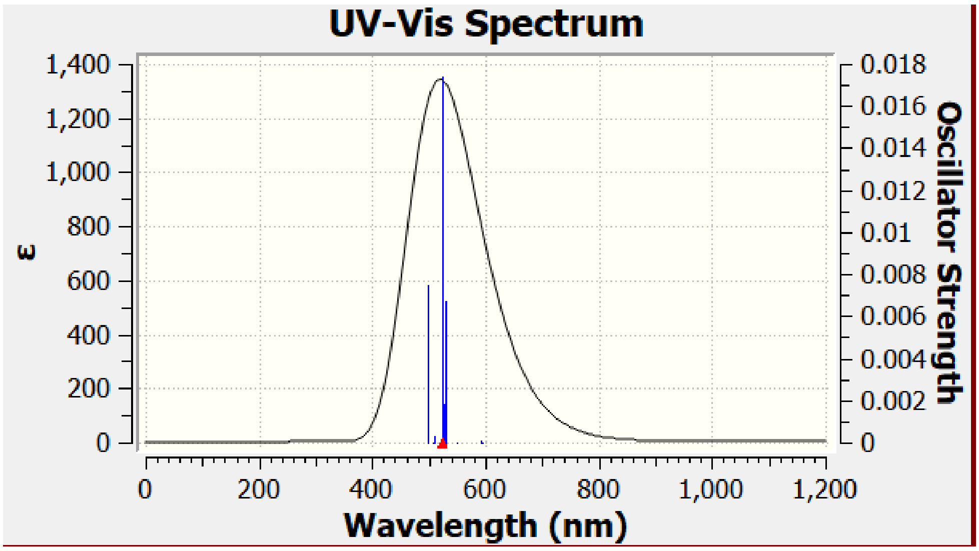

Figure S5. UV-VIS spectrum of $Co_2S_2(NO)_4$.

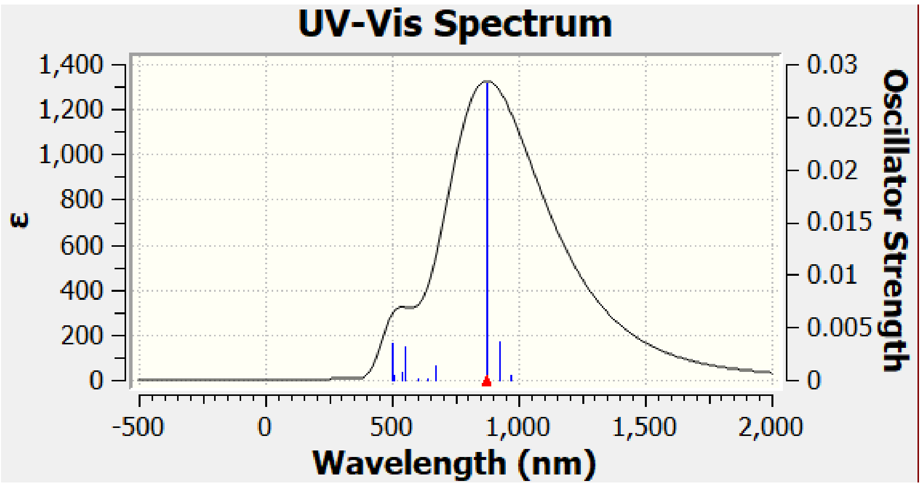

Figure S6. UV-VIS spectrum of $Co_2S_2(CO)_6(OH)$ with S-OH bond.

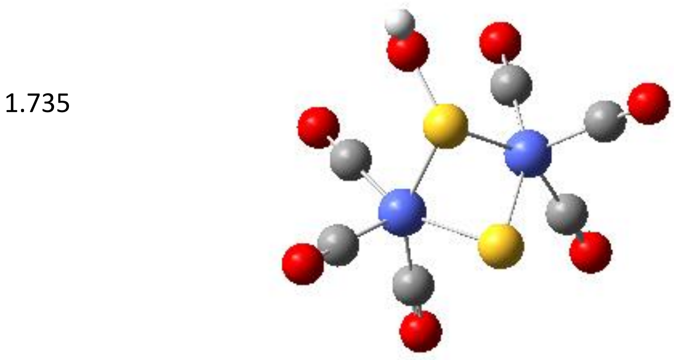

Figure S7. The binding of OH• group to $Co_2S_2(CO)_6$. Legend as Figure S3.

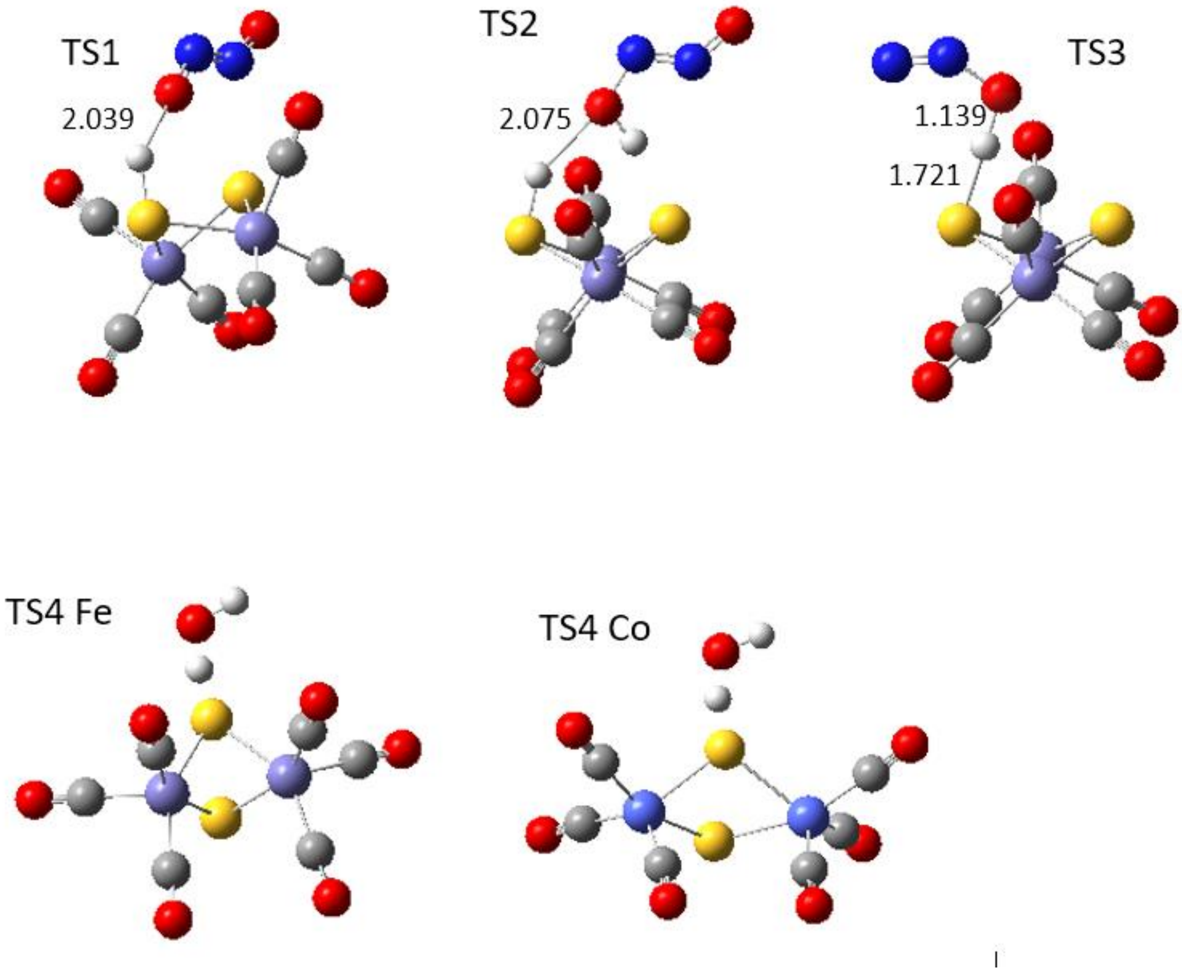

Figure S8. Transition states: TS1, TS2 and TS3 for Fe2S2 complexes; TS4 (water molecule release) for Fe2S2 and Co2S2, as denoted. Legend as Figure 6. Coordinates given below.

TS1 Co2S2 Complex
Co,O,-O.8O93117862,1.3728692691,O.2359447521
S,O,O.1673872772,O.193479OO43,1.9973642867
C,O,-1.8O83914819,1.5631472736,-1.269O447258
Co,O,-O.3636953873,-1.271O133144,O.258778O453
C,O,-2.17928O9257,1.75O598471,1.37O7228149
C,O,O.2726691662,2.9445819884,O.2952OO6874
S,O,O.4794O89912,O.217692O933,-1.2518355726
O,O,-2.447782448,1.6945939228,-2.1978441683
C,O,-1.2458534865,-1.8OO981338,-1.2378387864

C,0,-1.5337952886,-2.0603828881,1.4050955755
C,0,1.17378354,-2.3993483958,0.3371645089
O,0,-3.0380244977,1.9838941761,2.0732354968
O,0,0.9570267147,3.8434914887,0.3037266132
O,0,-1.8071516392,-2.1482256603,-2.1615133758
O,0,-2.2683734629,-2.5522195133,2.1149717541
O,0,2.1143905553,-3.0249863764,0.3550024691
N,0,3.2398357274,0.6675448486,-1.9544709002
N,0,3.5115361775,0.7224429104,-0.7448077424
O,0,3.8120558141,0.7524045938,-3.0118481953
O,0,2.7487155076,0.6062236334,0.2084102819
H,0,1.4829094829,0.4133250628,1.7901673709

TS1 Fe2S2 Complex
Fe -0.84427 1.19454 0.
S 0.41112 0.09511 1.50748
C -2.04272 1.47021 -1.36025
Fe -0.56177 -1.26019 -0.00046
C -1.98653 1.47599 1.40675
C 0.22676 2.66959 -0.02507
S 0.34854 0.08849 -1.55297
O -2.76971 1.62706 -2.21559
C -1.66634 -1.80038 -1.36086
C -1.6102 -1.79425 1.40613
C 0.81632 -2.45339 -0.026
O -2.67809 1.63598 2.29039
O 0.93028 3.5555 -0.0413
O -2.33873 -2.11794 -2.21629
O -2.24733 -2.10744 2.28967
O 1.70268 -3.15633 -0.04249
N 3.71128 0.88424 -1.75392
N 4.18675 0.86065 -0.61333
O 4.24821 0.98556 -2.83968
O 3.2274 0.72913 0.37775
H 0.04107 -0.06794 2.79864

TS2 Fe2S2
Fe,0,-0.5436571978,-1.3953118817,-0.4443282922
C,0,0.4054684928,-2.7228608623,-1.2172146276
C,0,-0.6141587292,-2.1268467637,1.2157691558
O,0,1.016954931,-3.5429010155,-1.7100585732
C,0,-2.2206454456,-1.982491359,-0.8728453504
S,0,0.8993109693,0.0534729289,0.4564435478
S,0,-0.630633379,-0.0189519913,-2.3184923991
O,0,-0.6491834741,-2.5847632278,2.2544001927
O,0,-3.2667150569,-2.3421226875,-1.1323006282
Fe,0,-0.8075026373,1.2363827937,-0.3676747125
C,0,-0.1820422097,2.7785583424,-1.0697740051
C,0,-1.0016007664,1.8486037253,1.3303820764
C,0,-2.5769798828,1.4767668775,-0.7537543509
O,0,0.2178734811,3.7418494795,-1.5188806223
O,0,-1.1137953944,2.233958622,2.3927139113
O,0,-3.6803432597,1.618628663,-0.98508956
O,0,2.6332479228,0.2531403569,-1.9153527498

H,O,2.2O61333199,O.2O29O34O61,-O.9967391193
H,O,O.68O382O313,O.1126943O22,-2.6O16494O33
N,O,4.3375983815,O.374O552158,-O.558778O35
N,O,3.97795O3196,O.3651942O57,-1.7492641337
O,O,5.43O27O4434,O.4615988298,-O.O459938851

TS2 Co2S2
Co -O.68362 -1.5O263 O.O216
C O.83O43 -2.41718 -O.14338
C -1.38629 -2.5OO61 1.38866
O 1.81593 -2.97219 -O.24O65
C -1.59167 -2.37188 -1.31689
S -O.2315 -O.OO438 1.57738
S -O.24953 O.O4854 -1.54199
O -1.82191 -3.1O98 2.24O87
O -2.16515 -2.88658 -2.1496
Co -1.12868 1.33668 O.O7352
C O.O3118 2.674O1 -O.O653
C -2.O9OO5 2.O2764 1.47242
C -2.27429 1.924O9 -1.2358
O O.8OO75 3.5O49 -O.14775
O -2.683O3 2.447O8 2.34398
O -2.98774 2.261O8 -2.O51O9
O 3.16633 1.11O61 -1.5OO41
H -O.915O2 O.21226 -2.7O858
N 4.98133 1.15O21 -2.57727
N 4.O5912 1.74668 -2.36O1
O 5.35O85 O.66266 -3.59O16

Hydroxy- intermediates, Cobalt complex
Co,0,1.3610038238,-0.007102431,-0.0949395213
C,0,1.6428347882,1.3073761608,1.1304461104
C,0,1.7265813458,-1.4237313729,0.986231782
O,0,1.8080682422,2.143515725,1.8801264665
C,0,2.9021033698,0.1181183817,-1.214355136
S,0,0.0195983044,-1.1000825076,-1.5118546828
O,0,1.9545481987,-2.3061874768,1.6609388098
O,0,3.7589010668,0.2075371184,-1.9484736767
Co,0,-1.3612608303,-0.0132513359,-0.1285666689
C,0,-1.680642538,1.3003961376,1.0884791999
C,0,-1.7457341856,-1.4317229947,0.9435413195
C,0,-2.8749527187,0.1037929133,-1.2856422891
O,0,-1.8691114326,2.1361221989,1.8331653508
O,0,-1.985278786,-2.3155379287,1.6124590594
O,0,-3.7142813361,0.1877897939,-2.0403347158
S,0,0.0098189506,1.4639169941,-1.1983387634
H,0,0.0289987519,-3.2740552515,-1.916650097
O,0,0.0187535351,-2.7819555045,-1.0856858273

Hydroxy intermediates, Fe-complex
Fe,0,-1.4852230384,0.2063200065,-0.235350254
C,0,-2.6147207192,-0.1739992125,1.1319705304
C,0,-2.2767597942,1.8074022743,-0.6710113499

O,0,-3.2908774968,-0.4213593824,2.008124042
C,0,-2.3363973094,-0.8892268087,-1.4214325328
S,0,0.000315246,-1.403402376,0.5208268018
S,0,-0.0003942235,1.1333000353,1.2758013952
O,0,-2.7128963818,2.818480642,-0.9376220557
O,0,-2.8319354355,-1.5978802661,-2.1575721163
Fe,0,1.4848360388,0.2071946387,-0.2354840201
C,0,2.6147009507,-0.1723223067,1.1317435211
C,0,2.2754042601,1.808695165,-0.671369403
C,0,2.3365323364,-0.8880006296,-1.4215187379
O,0,3.290923798,-0.4195175987,2.0078938884
O,0,2.7107992864,2.8200387971,-0.9381851523
O,0,2.8324391333,-1.5965041595,-2.157553938
O,0,-0.0003843336,0.6559811911,-1.5212086086
H,0,-0.0002709873,0.0867738402,-2.2963042102

Hydroxy intermediate a) iron complex
Fe,0,-1.4852230384,0.2063200065,-0.235350254
C,0,-2.6147207192,-0.1739992125,1.1319705304
C,0,-2.2767597942,1.8074022743,-0.6710113499
O,0,-3.2908774968,-0.4213593824,2.008124042
C,0,-2.3363973094,-0.8892268087,-1.4214325328
S,0,0.000315246,-1.403402376,0.5208268018
S,0,-0.0003942235,1.1333000353,1.2758013952
O,0,-2.7128963818,2.818480642,-0.9376220557
O,0,-2.8319354355,-1.5978802661,-2.1575721163
Fe,0,1.4848360388,0.2071946387,-0.2354840201
C,0,2.6147009507,-0.1723223067,1.1317435211
C,0,2.2754042601,1.808695165,-0.671369403
C,0,2.3365323364,-0.8880006296,-1.4215187379
O,0,3.290923798,-0.4195175987,2.0078938884
O,0,2.7107992864,2.8200387971,-0.9381851523
O,0,2.8324391333,-1.5965041595,-2.157553938
O,0,-0.0003843336,0.6559811911,-1.5212086086
H,0,-0.0002709873,0.0867738402,-2.2963042102

TS3 Co2S2
Co,O,-O.9157378817,1.3378291592,O.23O7633936
S,O,O.15249O7117,O.13O6984785,1.91611O3215
C,O,-1.876499OOO2,1.5124317167,-1.3OO81O8O56
Co,O,-O.5639997494,-1.3O5463678,O.2216O8O186
C,O,-2.294238858,1.6848366936,1.3615576899
C,O,O.14327O4198,2.9135OOOO18,O.29OO7O4511
S,O,O.414166249,O.1754976924,-1.2O69815188
O,O,-2.4926783338,1.63898OO768,-2.2473962372
C,O,-1.4619758961,-1.712O567284,-1.3O35845959
C,O,-1.788O865725,-2.O189451O25,1.3568O54572
C,O,O.8768862318,-2.541337O586,O.2578393962
O,O,-3.16O8497214,1.9O2O74OO42,2.O61397O49
O,O,O.837494OO8,3.8O75219431,O.2955394549
O,O,-2.O349633674,-1.9836665869,-2.2467546558
O,O,-2.5593749785,-2.4644517OO4,2.O6O1875842
O,O,1.7833259281,-3.219187O825,O.2462286696

N,0,4.3560716211,0.8050692706,-1.4971025667
N,0,4.0529168802,0.6996947621,-0.4249083077
O,0,3.7361076399,0.5906273671,0.7127062033
H,0,1.4207583692,0.2934761711,1.4902230586

TS3 Fe2S2
Co,0,-0.8093117862,1.3728692691,0.2359447521
S,0,0.1673872772,0.1934790043,1.9973642867
C,0,-1.8083914819,1.5631472736,-1.2690447258
Co,0,-0.3636953873,-1.2710133144,0.2587780453
C,0,-2.1792809257,1.750598471,1.3707228149
C,0,0.2726691662,2.9445819884,0.2952006874
S,0,0.4794089912,0.2176920933,-1.2518355726
O,0,-2.447782448,1.6945939228,-2.1978441683
C,0,-1.2458534865,-1.8000981338,-1.2378387864
C,0,-1.5337952886,-2.0603828881,1.4050955755
C,0,1.17378354,-2.3993483958,0.337164508
O,0,-3.0380244977,1.9838941761,2.0732354968
O,0,0.9570267147,3.8434914887,0.3037266132
O,0,-1.8071516392,-2.148225603,-2.1615133758
O,0,-2.2683734629,-2.5522195133,2.1149717541
O,0,2.1143905553,-3.0249863764,0.3550024691
N,0,3.2398357274,0.6675448486,-1.9544709002
N,0,3.5115361775,0.7224429104,-0.7448077424
O,0,3.8120558141,0.7524045938,-3.0118481953
O,0,2.7487155076,0.6062236334,0.2084102819

TS4 Co2S2 Complex
Co,0,1.83229799,0.0070588605,-0.1469846249
C,0,1.6731050895,1.6143611448,-0.9812601064
C,0,3.0159268985,0.5883742824,1.0677581976
O,0,1.6821603513,2.5838650016,-1.5753529121
C,0,2.9084515569,-1.3339550818,-0.755922027
S,0,-0.0021498192,-0.5588742314,1.1865209907
O,0,3.7373772877,0.9500007339,1.8632517226
O,0,3.5917460208,-2.1217204621,-1.2071265466
Co,0,-1.8675475565,0.0129564386,-0.1586608692
C,0,-1.6407414304,1.6161436464,-0.9821969779
C,0,-3.0827250218,0.6245058974,1.0022467551
C,0,-2.9263752808,-1.3440858844,-0.7564104438
O,0,-1.6032143512,2.5852115355,-1.5747370723
O,0,-3.8249701423,1.004272725,1.7724070011
O,0,-3.5984527191,-2.1429566195,-1.2044788623
S,0,-0.0209963032,-1.0419020188,-1.0032978715
H,0,-0.6147844697,-0.5094113016,3.7980468677
O,0,0.2100026851,-0.7422516716,3.3508165824
H,0,0.0096774985,-1.6369616208,2.0824886169

TS4 Fe2S2 Complex
Fe,0,1.3118649573,0.1198190186,-0.0196391083
C,0,2.9389658871,-0.1686865817,-0.7721650083
C,0,1.5533061715,1.8451102883,0.4450200808

O,O,3.96O5444314,-O.34O6682639,-1.2395O69196
C,O,1.6346194685,-O.538O9O4281,1.63692O3648
S,O,O.OO57512351,-1.458O4O7667,-O.8464231437
S,O,O.OO26O98893,O.4854165127,-1.8741919O2
O,O,1.7116549O75,2.94OOOO5654,O.7131O698
O,O,1.834716O482,-O.96133O6375,2.672923388
Fe,O,-1.3179546163,O.114O464385,-O.O278768338
C,O,-2.9433256561,-O.17O42O87O5,-O.783O3228O5
C,O,-1.5524731442,1.84224O6358,O.426O413668
C,O,-1.6276695349,-O.5169376931,1.637O842962
O,O,-3.967O82577,-O.34O3971784,-1.2484538644
O,O,-1.7O87762O84,2.939186O264,O.687OO56432
O,O,-1.8234848738,-O.927O323576,2.68O8727682
O,O,O.1139457617,-3.4219OO7O36,-O.1341O8O877
H,O,-O.73O754O465,-3.72234OO6O3,O.2292443142
H,O,-O.OO473117O1,-2.68117O3243,-1.5338302439

References

1. Becke, A.D. Density-functional thermochemistry. IV. A new dynamical correlation functional and implications for exact-exchange mixing. *J. Chem. Phys.* **1996**, *104*, 1040.
2. Becke, A.D. Density-functional thermochemistry. *III. The role of exact exchange. J. Chem. Phys.* **1993**, *98*, 5648.
3. Lee, C.; Yang, W.; Parr, R.G. Development of the Colle-Salvetti correlation-energy formula into a functional of the electron density. *Phys. Rev. B* **1988**, *37*, 785.
4. Miehlich, B.; Savin, A.; Stoll, H.; Preuss, H. Results obtained with the correlation energy density functionals of Becke and Lee, Yang and Parr. *Chem. Phys. Lett.* **1989**, *157*, 200.
5. Wachters, A.J.H. Gaussian basis set for molecular wavefunctions containing third-row atoms. *J. Chem. Phys.* **1970**, *52*, 1033.
6. Hay, P.J. Gaussian basis sets for molecular calculations. The representation of 3d orbitals in transition-metal atoms. J. Chem. Phys., **1977**, *66*, 4377.
7. Raghavachari, K.; Trucks, G.W. Highly correlated systems. Excitation energies of first row transition metals Sc–Cu. J. Chem. Phys., **1989**, *91*, 1062.
8. Hay, P.J.; Wadt, W.R. Ab initio effective core potentials for molecular calculations. Potentials for the transition metal atoms Sc to Hg. *J. Chem. Phys.* **1985**, *82*, 270.
9. Halgren, T.A.; Lipscomb, W.N. The synchronous-transit method for determining reaction pathways and locating molecular transition states. *Chem. Phys. Lett.* **1977**, *49*, 225.
10. Peng, C.; Ayala, P.Y.; Schlegel, H.B.; Frisch, M.J. Using redundant internal coordinates to optimize equilibrium geometries and transition states. *J. Comp. Chem.* **1996**, *17*, 49.
11. Fukui, K. The path of chemical reactions-the IRC approach. *Acc. Chem. Res.* **1981**, *14*, 363.
12. Hratchian, H.P.; Schlegel, H.B. Accurate reaction paths using a Hessian based predictor–corrector integrator. *J. Chem. Phys.* **2004**, *120*, 9918.
13. Bauernschmitt, R.; Ahlrichs, R. Stability analysis for solutions of the closed shell Kohn–Sham equation. *J. Chem. Phys.* **1996**, *104*, 9047.
14. Bauernschmitt, R.; Ahlrichs, R. Treatment of electronic excitations within the adiabatic approximation of time dependent density functional theory. *Chem. Phys. Lett.* **1996**, *256*, 454.
15. Furche, F.; Ahlrichs, R. Adiabatic time-dependent density functional methods for excited state properties. *J. Chem. Phys.* **2002**, *117*, 7433.
16. Yanai, T.; Tew, D.; Handy, N. A new hybrid exchange-correlation functional using the Coulomb-attenuating method (CAM-B3LYP). *Chem. Phys. Lett.* **2004**, *393*, 51–57. https://doi.org/10.1016/j.cplett.2004.06.011.
17. Reed, A.E.; Curtiss, L.A.; Weinhold, F. Intermolecular interactions from a natural bond orbital, donor-acceptor viewpoint. *Chem. Rev.* **1988**, *88*, 899.
18. Weinhold, F.; Carpenter, J.E. *The Structure of Small Molecules and Ions*; Plenum: New York, NY, USA, 1988).
19. Tomasi, J.; Mennucci, B.; Cammi, R. Quantum Mechanical Continuum Solvation Models. *Chem. Rev.* **2005**, *105*, 2999–3093. https://doi.org/10.1021/cr9904009.
20. Grimme, S.; Ehrlich, S.; Goerigk, L. Effect of the damping function in dispersion corrected density functional theory. *J. Comp. Chem.* **2011**, *32*, 1456–1465. https://doi.org/10.1002/jcc.21759.

.
